# Bariatric Bypass Surgery to Resolve Complicated Childhood Morbid Obesity: Case Report Study

**DOI:** 10.1097/MD.0000000000002221

**Published:** 2015-12-11

**Authors:** Abduh Elbanna, Mohammed Tag Eldin, Mohammad Fathy, Osama Osman, Mohammed Abdelfattah, Abdelrahman Safwat, Mohammed Sedki Abd Elkader, Shymaa E. Bilasy, Khaled salama, Asim A. Elnour, Abdullah Shehab, Shazly Baghdady, Mohamed Amer, Mohamed Alboraie, Aly ragb, Abd Elrazek Abd Elrazek

**Affiliations:** From the Laparoscopic and Bariatric Surgery Department, Al Azhar School of Medicine, Al Hussein University Hospital (AE, M Amer, OO, MF, MTE, AY); Pediatric Department, Misr International School of Medicine, Cairo (MAE); Biochemistry Department, Faculty of Pharmacy, Suez Canal University, Ismailia (SB); Internal Medicine Department, Al Azhar school of Medicine, Asuit, Egypt (KS); Cardiovascular Medicine and Pharmacology Departments, Faculty of Medicine and Health Sciences, United Arab Emirates University, United Arab Emirates (AAE, AS); Pulmonology and ICU Departments, Aswan School of Medicine, Aswan University, Aswan (SB); Hepatology Department, National Liver Institute, Minoufiya University, Minoufiya (M Amer); Internal Medicine Department, Al Azhar school of Medicine, Cairo, Egypt (M Alboraie); General and Laparoscopic Surgery Department, Al Azhar School of Medicine, Al Hussein University Hospital, Cairo (AR); and GIT & Hepatology Department, Al Azhar School of Medicine, Asuit Branch, Asuit, Egypt (AEAE).

## Abstract

Children obesity has become one of the most important public health problems in many countries worldwide. Although the awareness of childhood obesity as a modifiable health risk is high, but many societies do not prioritize this issue as a health care problem, which may lead to comorbidities and even premature death. Despite the rising interest in bariatric surgery for children, only laparoscopic sleeve gastrectomy (LSG) is being considered in resolving childhood obesity who failed other dietary or drug therapies; however many of LSG procedures failed to reduce the weight in children or resulted in complications postsurgery.

Here, we present a novel bariatric procedure to clue out a female child 13 years old presented with Legg–Calvé–Perthes disease-associated morbid obesity. The surgical bariatric technique applied both fundal resection and surgical bypass in pediatric obesity using the Elbanna novel bariatric technique.

Bariatric surgical bypass may be considered in complicated-childhood cases who failed all other options.

## Abstract 2

Supplemental Digital Content is available in the text

## INTRODUCTION

Obesity, defined as a body mass index (BMI) ≥30 kg/m^2^, is a chronic illness identified in children, adolescents, and adults worldwide. According to the World Health Organization, worldwide there are ∼ 500 million obese adults and 42 million obese children under the age of 5.^1,2^ Childhood obesity became one of the most important public health problems in many countries with special concern to industrial countries. In the United States alone, reports dedicate comorbidities and premature death of children who have severe obesity. Therefore, it is imperative that health care providers should identify overweight and obese children to start early counseling and therapy.^3–5^ Until now there are no established guidelines for bariatric surgery in childhood morbid obesity with complications. Considering such surgical intervention in childhood is a dilemma of substantial debate; however, if diet, exercise, and drugs fail to control comorbidities-related obesity in children, bariatric surgical interventions should be suggested and the technique of choice should be optimized for each specific case.

### METHODS AND EXPLANATION

This case study was approved by the Ethics Committee of Al-Azhar School of Medicine, Egypt, and the patient and her family provided a written consent.

The preoperative assessment included pediatric, psychiatric, respirology, cardiology, orthopedic, and gynecology consultations, routine biochemical lab investigations (complete blood count [CBC], erythrocyte sedimentation rate [ESR], and C-reactive protein [CRP] plus hepatitis B and C virus testing, liver, kidney, and thyroid function tests, a lipid profile, fasting glucose and insulin, hemoglobin A1c, urinalysis, stool analysis, vitamin D levels, parathyroid hormone levels, and *Helicobacter pylori* testing. Ultrasonography and computed tomography of the liver and kidney showed marked fatty liver infiltration, indicating nonalcoholic fatty liver disease (NAFLD), although the liver enzymes were elevated only slightly.

## CASE REPORT

**FIGURE 1:**
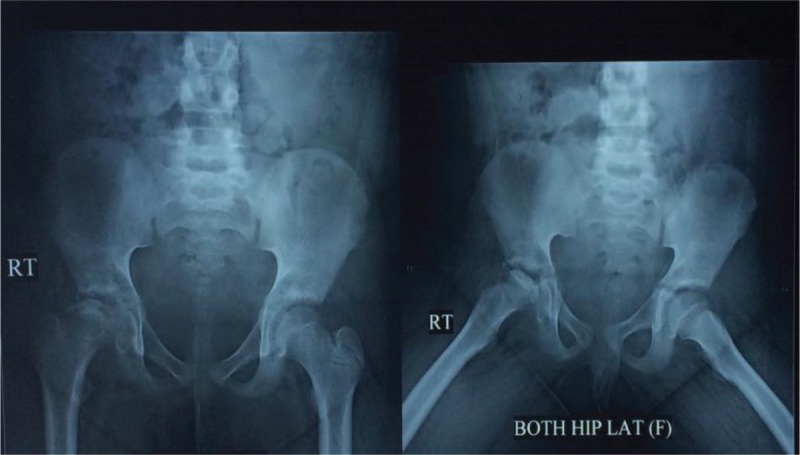
Both hip joints in standing and diversion positions. Note the right hip deformity with slipped femoral head; picture is suggestive of a vascular necrosis of Perthes disease.

**FIGURE 2:**
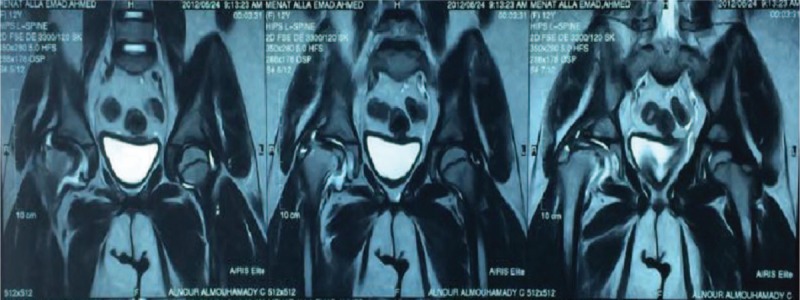
MRI of both hip joints. Note the right hip joint effusion, with deformed and collapsed right femoral head of altered signal intensity; picture is suggestive of high-grade Legg–Calvé–Perthes disease. MRI = magnetic resonance imaging.

A 13-year-old girl presented with Legg–Calvé–Perthes disease (avascular necrosis of her right hip joint) (Figs. 1 and 2), due to long-standing morbid obesity. Her body mass index (BMI) at presentation was 44.89 (weight, 88 kg; height, 140 cm). This was accompanied by NAFLD, depression, and secondary amenorrhea. A psychiatric consultation revealed childhood depression-related fatness and disapproval of her friends and schoolmates. A gynecological consultation revealed secondary amenorrhea related to obesity, induced by hormonal disorders. Both the psychiatrist and the gynecologist recommended weight reduction by any means. An orthopedic surgeon recommended hip joint replacement for the diseased hip, and advised weight reduction so as to not affect the other hip, remarking that the condition was serious at such an early age. All dietary, lifestyle, and medication measures failed to resolve her condition. Although a dietician scheduled the patient for a combined exercise and diet regimen, the orthopedic surgeon recommended that the exercises be discontinued so as not to affect the other hip and aggravate the diseased hip. The patient often resorted to eating when feeling depressed.

**FIGURE 3:**
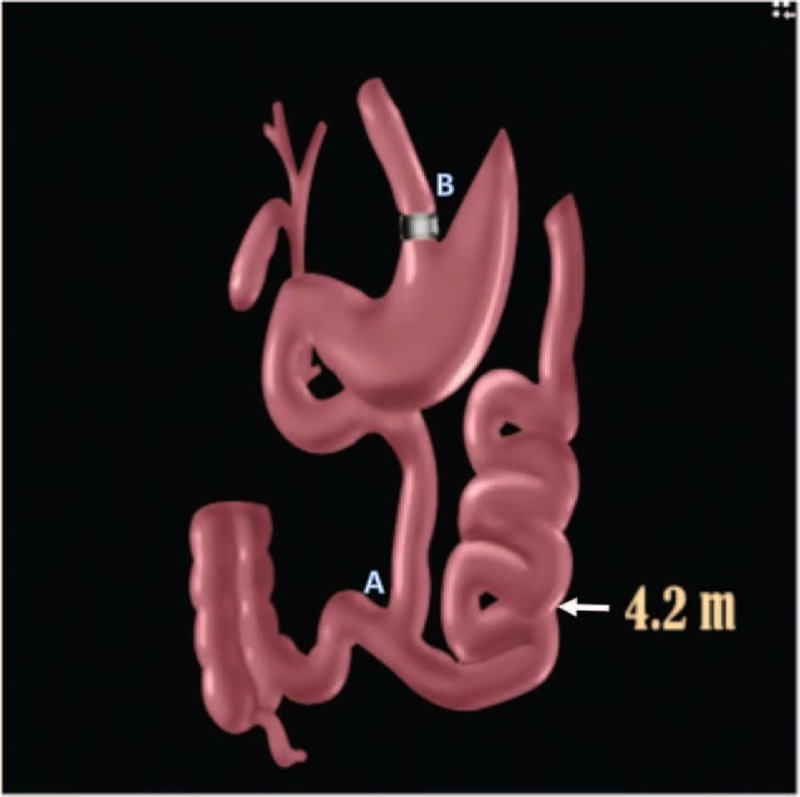
Novel Elbanna Procedure: at 50 cm from the dudenojejunal flexure we transect the jejunum. Reanastomosis (A) is performed between the proximal jejunum and the terminal ileum, 100 cm from the ileocecal valve. Bypass 4.2 m of the jejunum. Duodenum, proximal 50 cm of jejunum, and 100 cm of terminal ileum help the physiological absorption. Fundal resection (B) making banded pouch is performed to get maximum effect on appetite and satiety. Preservation of the anatomical biliary drainage and enterohepatic circulation are the most procedural advantage.

Given the reported weight regain after long-term follow-up of laparoscopic sleeve gastrectomy (LSG) patients and the general tendency for those with a family history of obesity to regain weight after dietary or standard bariatric therapies, we decided to perform the Elbanna operation in our patient given the high BMI (44.89), positive family history of adiposity, fatty liver, and avascular necrosis of her hip joint. This novel procedure, a modified jejunoileal anastomosis with fundal resection (Fig. 3), has been performed successfully on 196 adults in Al Azhar University Hospital, Cairo, Egypt. The treated patients successfully regained a normal BMI without suffering from malabsorptive side effects with fewer complications than other bariatric procedures.

This novel intervention preserves the biliary anatomy compared to other bypass surgeries and results in a significant improvement in liver function compared with a standard sleeve gastrectomy. The anatomical, surgical, and physiological concept behind the novel Elbanna procedure is to preserve the gastrointestinal anatomy where most of the digestive enzymes, HCl, hormones, and intrinsic factors are secreted, as far as possible. Furthermore, the absorption of digested amino acids and biliopancreatic enzymes is essential for the digestion of protein and fats to extract vitamins, which are important during childhood. Both vitamins and minerals are absorbed in the preserved segments.

First, we transect the jejunum 50 cm from the duodenojejunal flexure. A reanastomosis is performed between the proximal jejunum and terminal ileum 100 cm from the ileocecal valve. The duodenum, proximal jejunum, and terminal ileum help maintain physiological absorption. Preservation of the anatomical biliary drainage and enterohepatic circulation are the greatest procedural advantages. Fundal resection is performed to obtain the maximum effect on appetite and satiety.

**FIGURE 4:**
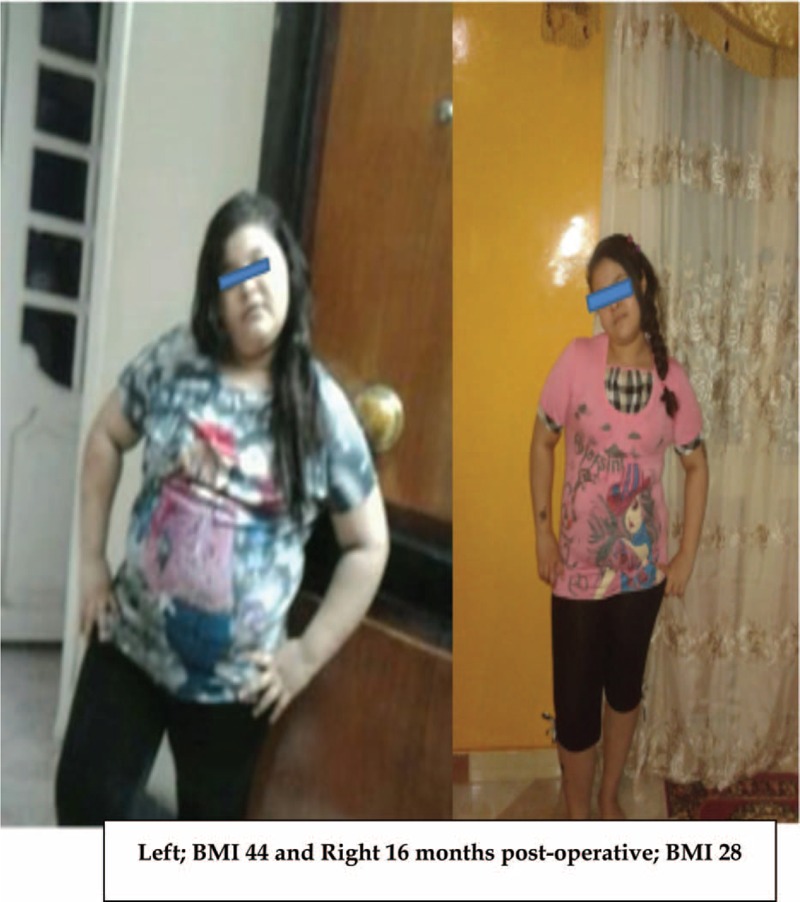
The female child preoperative: (left) BMI = 44 and (right) 16 months postoperative with BMI = 28. BMI = body mass index.

Two months after the clinical evaluation and after considering the ethical issues, the Elbanna operation was performed on the child. The operating time was 100 min. The gastrografin test was successful at the end of the procedure and 3 days postoperatively. The child did not need a blood transfusion during or after the operation. She was discharged on the fourth postoperative day in the average general condition. No early or late postoperative complications were reported. Follow-up revealed progressive weight loss without the need for vitamin, mineral, or iron supplementation. Her estimated weight loss 4, 8, and 12 months postoperatively was 15, 26, and 32 kg, respectively. She underwent gradual psychological improvement and her biochemical parameters normalized. Five months postoperatively, the child experienced regular menstruation. Sixteen months postoperatively, her BMI was 28 with acceptable sonographic appearance of the liver and normal hepatic enzymes. The patient's psychological condition improved and she had a nice body contour (Fig. 4). She stated, “*Every time I look at my body in the mirror, I feel that I have been born again. I feel very happy playing with my friends now*.”

## DISCUSSION

The marked increase in the incidence of obesity in the past 30 years is due to several factors, including increased caloric intake (ie, fast food), changes in the composition of the diet (ie, flavored meals), a decrease in the levels of physical activity (ie, sedentary lifestyle), and changes in the gut microbiome.

Childhood obesity has become an important public health problem worldwide. In the United States, the highest prevalence of childhood obesity is in African–American and Hispanic populations, and these groups have significantly more cardiovascular risk factors and are at a greater risk of having obesity-associated comorbidities in adulthood; therefore, it is imperative that health care providers identify overweight and obese children so that counseling and treatment can be provided.^1^

The comorbidities of obesity in childhood include abnormalities in the endocrine, genitourinary, cardiovascular, psychosocial, hepatic, pulmonary, orthopedic, neurologic, and dermatologic that affect both the quality of life and survival. Treating childhood obesity means overcoming the illness at present, avoiding future complications in adulthood, and alleviating the patient's economic burden in the present and future. We should consider bariatric surgery if there is failure to control associated comorbidities in childhood, such as sleep apnea, hypoventilation syndrome, and bone or joint disorders (slipped femoral epiphysis, Legg–Calvé–Perthes, and tibia vara), and in children at increased risk of cardiovascular disease.^6–14^

A novel bariatric surgical technique, the Elbanna operation,^15–18^ shows promise in adulthood bariatrics. This technique is designed to maintain good digestion, better satiety, and selective absorption with fewer medical and surgical complications. The Elbanna procedure preserves the duodenum, proximal jejunum, and terminal ileum, along with the anatomical biliary drainage and enterohepatic circulation. In addition, fundal resection is performed to obtain the maximum weight loss. The Elbanna technique avoids the vitamin and trace element deficiencies that occur after other surgical bariatric diversion techniques, such as biliopancreatic diversion with or without duodenal switch, Roux en Y, mini gastric bypass, and sleeve bypass. The Elbanna technique also preserves biliopancreatic secretions, which are very important in growing children, as it preserves the anatomical external biliary pathway.

The old-fashioned jejunoileal bypass (JIB) is purely malabsorptive bariatric surgery that was popular in the 1960s and 1970s. The procedure results in significant weight loss by creating a surgical short bowel syndrome; however, JIB is no longer used today because of the 50% morbidity and 10% mortality rates.^19–26^ In comparison, the Elbanna-modified JIB technique preserves the proximal jejunum and terminal 100 cm of the ileum, so no short bowel syndrome or hepatic, renal, or metabolic complications have been reported. Interestingly, no vitamin or mineral supplements are required postoperatively. The cornerstone of the novel Elbanna technique is that it replaces the maldigestion and malabsorption concept of traditional bariatric procedures with a new concept of good digestion and selective absorption.

Unfortunately, there are no reports on the overall success rates or complications following bariatric bypass surgery in childhood obesity due to a lack of data. With the increased use of bariatric surgery to treat obesity in children, some guidelines have been published, most of which exclude children <14 years. Only a few reports describe LSG in children and adolescents, so its safety and effectiveness are not clear and no guidelines for its use have been developed.^27–28^ Currently, bariatric surgery to treat childhood obesity is passing through a plateau phase, and the medical management and follow-up of children who have undergone bariatric surgery is a challenge. Previously, we reported obese patients with Legg–Calvé–Perthes disease treated with the Elbanna operation. Here, we present the first bariatric bypass surgery performed in childhood obesity using the Elbanna technique with detailed, careful follow-up.

We strongly believe that bariatric surgery may be considered in complicated pediatric patients when all medical therapies have failed. In addition, the effects of depression and psychosocial disorders in obese children should be considered, so as not to affect their future.

## Supplementary Material

Supplemental Digital Content
