# Bariatric Bypass Surgery to Resolve Complicated Childhood Morbid Obesity: Case Report Study: Erratum

**DOI:** 10.1097/01.md.0000484063.75497.06

**Published:** 2016-05-20

In the article “Bariatric Bypass Surgery to Resolve Complicated Childhood Morbid Obesity: Case Report Study”^1^, which appeared in Volume 94, Issue 49 of *Medicine*, Dr. Elrazek's name was incorrectly presented as Abd Elrazek M. Ali Hussein when it should have read Abd Elrazek Abd Elrazek. The article has since been corrected online.
